# Some Novel Relations in Human Pathology

**Published:** 1920-05-08

**Authors:** 


### MAN AND THE HIGHER FUNGI.

### Some Novel Relations in Human Pathology.

WHEN we consider how small a section in our current medical text-books is devoted to disease directly caused by organisms of a higher order than the common bacteria, and yet not among the better known parasitic infections, such as malaria, bilharziasis, etc., it comes as a great surprise when we read Dr. Castellani's description in the recent Milroy lectures of the large number of ways in which *mycological* or "yeast" affections can occur in the human system. It is true that a high proportion of his cases and instances are drawn from tropical or sub-tropical experience, and that he admits the conditions commonly occurring in England in the past have been of relatively simple pathology, but there are two points in his address which should be noted by every practitioner in the country who cares to know the true cause of the conditions which he treats. The first is that a number of the curious sub-acute lesions which have occurred among troops returning from the Eastern war zones may have been due to pathological strains of organisms usually considered harmless in the common practice of this country, and which were, therefore, passed over in any laboratory examination made; and the second is that not only must we in future be on the look-out for such cases, but must also realise that mycotic disease-producing infections are, even in Europe, less simple and far more frequent than the old-time teaching would have us believe. Even with regard to so simple an ailment as "thrush" the author concludes that it is "not caused by one species of fungus only, the so-called thrush fungus or *Oidium Albicans*, as generally stated. It is caused by a number of different fungi, some of which are botanically very far apart from each other, and belong to separate species, genera and families."

### BRONCHO-MYCOSES.

It is not so much, however, with the well-known and exhaustive laboratory differentiations which Dr. Castellani has made that the practising physician has to deal. Rather must he be on the look-out for the habit which these fungoid infections have of imitating sundry of the classical pictures of specifically recognised disease. Take, for example, the possible bronchial infections, "broncho-mycoses," and their possible confusion with tuberculosis. The fallacy has been perhaps better recognised in the case of domestic animals, but man is by no means exempt. Bronchial infection and extension into the lung substance can occur from a variety of species, and cases have been noted in this country relatively often. In some cases there is an occupational cause, such as tea-taster's cough—a broncho-moniliasis due to inhaling spore-containing tea-dust. A case is described of infection in a groom by *mucor mucedo*, a common organism in debris of the stable, and in France a peculiar aspergillosis is common among pigeon-breeders, who fill their mouths with grain before blowing it into the mouths of the pigeons.

In such infections the diagnosis is generally to be made from phthisis, and the absence of the tubercle bacillus, together with a profusion of yeast-like bodies in the sputum, is usually the first differential indication. The severity of the ultimate result depends largely upon the variety of fungus present. The milder types, in which recovery is practically the invariable rule, need no special description, but the severer varieties are extraordinarily similar to acute tuberculous extension. The patient becomes emaciated, there is hectic fever, and expectoration frequently contains blood, while on examination the chest may show patches of dullness, crepitations, and pleural rubbing. Treatment is again according to the type of fungus responsible; and let us note that potassium iodide, though practically specific for certain species, has apparently no effect whatever upon others. In this type of acute bronchial infection there is, indeed, a condition which has received little enough consideration in the medical teachings of this country.

### TONSILLO-MYCOSES

Of a far less sinister meaning, but nevertheless of great practical interest, is the frequency with which mycotic infections of the tonsils, palate, &c., simulate the local lesions in diphtheria. Complete laboratory examination should immediately establish the diagnosis, but even a direct film of the sputum will provide much information, particularly in the limited circumstances so frequently accompanying work in the East. Especially is this important owing to the possibility of rapid extension to a broncho-mycosis, as described in the previous section, if careful local treatment is omitted, and reliance placed on the specific effect of an anti-diphtheritic serum. When the mycotic conditions possibly found in an adult tonsil are appreciated, it requires no stretch of imagination to predict that frequently these fungi may be the *root* cause of a variety of nasal and aural conditions, although pyogenic cocci, &c., may flourish at the same time. So simple a condition as mild rhinorrhœa, or ultimately an acute mastoiditis, may find its actual cause in some fungoid growth. Dr. Castellani describes a case of repeated attacks of severe sneezing, due absolutely to a nasal infection with *aspergillus niger*, and it is unnecessary to enlarge on the potential power of secondary coccal infections arising in such cases.

### THE GENITO-URINARY SYSTEM.

Similarly, attention is drawn to another possible focus, namely, the genito-urinary organs. Urethritis of a hyphomycetic or mycotic origin is proved to be a definite clinical entity as far as the Near East is concerned, and even in this country we have been fortunate in seeing cases the history of which

**Man and the Higher Fungi—(*continued*).**

could be dated to travels in those regions, and probably corresponded in their early stages to Dr. Castellani's types. Fortunately, efficient local treatment in the male rapidly reduces such infections, the characters of which, after the first short period, are in marked contrast to any of the simple venereal or pseudo-venereal infections commonly seen in England; but turning to the question of female cases, we see a greater possibility, from anatomical reasons, that mycotic infection, vaginal in the first place, may be a relatively frequent condition. Apparently, in the tropics, the mycotic infection in either sex is by no means uncommon, and the author and others have drawn frequent attention to them in England. It is too long a subject to discuss at the moment, but the actual possibility of "yeasts" being infecting agents of the genito-urinary system, of their simulating venereal diseases, and even of being transmitted by sexual intercourse, is a point of significance of both the medical and legal aspect of these problems.

DISEASES OF THE SKIN.

From a purely scientific aspect, the most interesting pathological lesions caused by the higher fungi can be classed among diseases of the skin. To quote Dr. Castellani, "It is interesting to note that, though, of course, during recent years the all-important *rôle* played by the higher fungi in dermatology has been amply recognised, not many decades ago most authorities denied them any importance, some considering such organisms to be mere saphrophytes, and others going so far as to state that the so-called fungi found in the epidermis and the hairs did not exist, these structures merely representing a granular degeneration of the epidermal cells." The now accepted views on fungoid skin lesions are perhaps the best recognised of those dealt with in this note and to those who wish to follow the paths somewhat further than standard dermatological works can take them, no better reference can be given than these Milroy lectures which the *Lancet* has lately published. Taking, on the other hand, a general view, and summarising the impression which Dr. Castellani's words leave, the reader—particularly if he be something of a pathologist by inclination—cannot fail to appreciate how often in the routine work of both the clinic and the laboratory we may miss the significance of some simple "yeasts," or at least fail to give due consideration to their possible pathogenic power. There is no doubt that these fungi are far from being the necessarily harmless or adventitious organisms which many of us have been accustomed to believe—and we should do well to further such investigational work as the author has so ably begun.

